# How might Hospital at Home enable a greener and healthier future?

**DOI:** 10.1038/s41746-024-01249-8

**Published:** 2024-09-16

**Authors:** Dylan Powell, Fanny Burrows, Geraint Lewis, Stephen Gilbert

**Affiliations:** 1 Faculty of Health Sciences & Sport, University of Stirling, Stirling, UK; 2 NHS England London, London, UK; 3 Microsoft Corporation, 2 Kingdom Street, London, UK; 4 Else Kröner Fresenius Center for Digital Health, TUD Dresden University of Technology, Dresden, Germany

## Abstract

Traditional healthcare delivery models face mounting pressure from rising costs, increasing demand, and a growing environmental footprint. Hospital at Home (HaH) has been proposed as a potential solution, offering care at home through in-person, virtual, or hybrid approaches. Despite focus on expanding HaH provision and capacity, research has primarily explored patient care outcomes, patient satisfaction economic costs with a key gap in its environmental impact. By reducing this evidence gap, HaH may be better placed as a positive enabler in delivering healthier planet and population. This article explores the environmental opportunities and challenges associated with HaH compared to traditional hospital care and reinforces the case for further research to comprehensively quantify the environmental impact including any co-benefits. Our aim for this article is to spark conversation, and begin to help prioritise future research and analysis.

Health systems worldwide face significant challenges to population health, including the increased multimorbidity in an ageing population and spiralling delivery costs. This combination of challenges has prompted closer dialogue and demand for innovative and resilient approaches to healthcare delivery^1^. One proposed solution inpartly combatting these challenges is expansion of Hospital at Home (HaH), often defined as the provision of inpatient-level care at home, regardless of whether that care is delivered in person, through digital/virtual approaches, or a hybrid of the two^2,3^. Despite the significant focus on expanding HaH provision and capacity, in particular during the COVID-19 pandemic, barriers remain to widespread adoption. A recent perspective in *npj Digital Medicine* exploring the future of HaH also found several hurdles to overcome, including finance models, workforce and care delivery models^2^. With the World Health Organisations (WHO) describing climate change as the 21^st^ century’s greatest threat to global health, the environmental contribution and impacts of new models of care are increasingly required to be considered in parallel. Of note the evidence base for HaH has prioritised focus on patient care outcomes^2,4,5^, patient preference and economic costs^4^ with the environmental considerations being less discussed. Therefore, there is demand to evaluate and explore the costs and benefits to both public health and environmental sustainability that HaH may provide. This also leads to a key question, what steps might be required to enable HaH to better concurrently progress both planetary and population health? In this News and Views article we explore the potential environmental challenges and opportunities associated HaH with recommendations for future research.

## What do we mean by Hospital at Home?

**Fig. 1:**
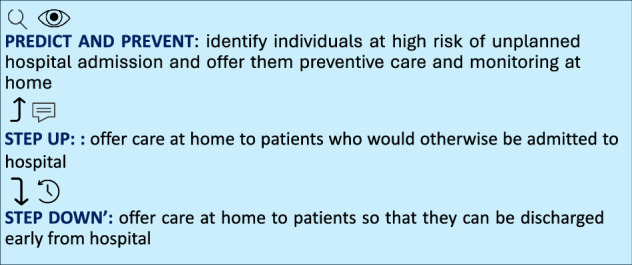
Functions and approaches in Hospital at Home. Typical functions and approaches in Hospital at Home (HaH), Predict and Prevent, Step Up, and Step Down. Parts of this figure utilizes icons obtained via the Noun Project all licensed under CC BY 3.0 License.

For the purpose of this article, we consider the definition of HaH in the broadest sense, namely the provision of inpatient-level care at home, regardless of whether that care is delivered in-person, through digital/virtual approaches, or a hybrid of the two (Fig. 1). Despite HaH not being a new concept the definitions and scope of HaH vary significantly, making comparisons difficult locally and internationally. Even with significant variation, in the UK, the concept of HaH appears broadly well accepted, with nearly three-quarters of the UK public (71%) saying they are open to being treated through HaH under the right circumstances^6^ and up to 63% of staff supportive of HaH as a concept^6^.

A recent assessment by The National Institute for Health and Care Excellence (NICE) found that HaH can be a safe and cost-effective compared to in-patient benchmarks^7^. This finding was primarily attributed to two factors: fewer patients occupying hospital beds (reduced bed days) and the lower daily cost of providing care at home compared to a hospital setting. It was, however, noted that many of the included studies had potential serious limitations in their methodological quality, with limited considerations of long-term outcomes and costs, including the environmental benefits or costs associated with HaH. Therefore, despite some encouraging findings, further economic, social and environmental evaluation of HaH is warranted^2^.

## Exploring the environmental barriers and opportunities for Hospital at Home

**Fig. 2:**
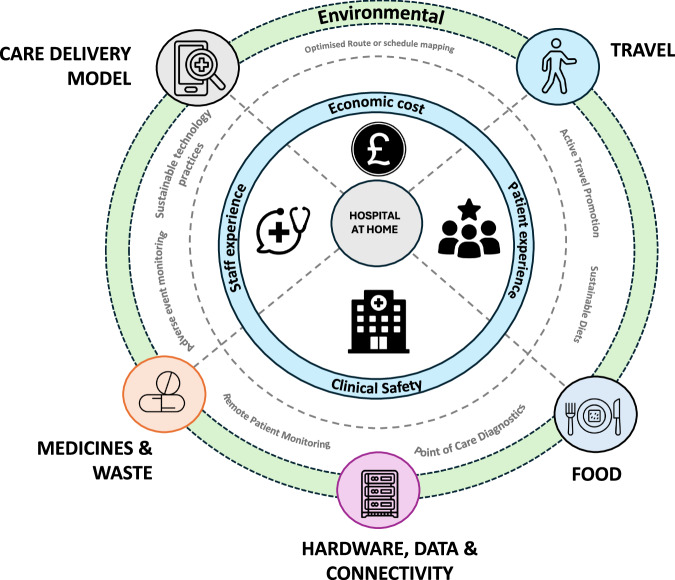
Environmental considerations of Hospital at Home. Exploring the key environmental considerations for Hospital at Home (HaH) including Care Delivery Model, Travel, Food, Hardware, Data, Connectivity and Medicines and Waste. Parts of this figure utilizes icons obtained via the Noun Project all licensed under CC BY 3.0 License.

Estimates suggest that if the global healthcare sector was a country, it would be the fifth largest emitter of greenhouse gases^8^. As such healthcare has a critical role in mitigating and adapting to the impacts of climate change. As a consequence, the NHS in England, responsible for around 4% of England’s total carbon footprint has set itself the ambitious target of becoming the world’s first net zero health system by 2045^9–12^. In this section, we highlight some important aspects that need careful attention in the pursuit of delivering more environmentally sustainable HaH (Fig. 2).

### Travel

In England, patients, visitors, staff, and suppliers travelling to NHS facilities contribute approximately 3.5% of all road travel, which accounts for about 14% of the health system’s total emissions^11,13^. This not only increases traffic volumes but also has a negative impact on air quality, resulting in significant environmental and health costs. Indeed, studies suggest that the air pollution caused by NHS-related travel leads to higher mortality and societal costs exceeding £345 million^14,15^. The promotion of active travel through cycling and walking has the potential to provide co-benefits for health and the environment.

As well as the electrification of transport, technology can help with route planning for staff when they travel^16^ and with patient support programmes for self-management and promoting community involvement^17,18^. Additional opportunities to reduce emissions exist in distributed health care, including offering multidisciplinary consultations and co-locating providers and allied health professionals to minimise patient travel. A broader paradigm shift of moving care closer to home will, however, require a detailed examination of the impacts of these changes including challenges such as equity of access and cost.

### Care delivery model

The environmental impact of health and social care has a significant influence on where and in what setting the care is delivered^19^. For example, the carbon footprint of an average GP appointment is 6 kg CO2e (rising to 18 kg CO_2_e with prescriptions) compared to the 708 kg CO_2_e associated with an elective inpatient stay^19^. Technology-enabled models of care, such as the use of telehealth, can increase capacity and support patients in ways that are more environmentally sustainable. However barriers remain in quantifying the environmental footprint of individual patient care pathways, which can make comparisons of alternative models difficult. There is an opportunity to move away from the estimation of carbon impacts to more standardisation and greater use of objective methods, calculations and real-world life cycle assessments.

### Medicines and waste

Medicines represent a significant burden on the environment and account for 25% of all emissions within the NHS^11^. It has been estimated that between one third and a half of patients on long term treatment outside of hospital do not take their medications correctly, with an estimated cost of €125 billion and 200,000 premature deaths in Europe each year^20–22^. As such, there is an opportunity to explore ways to improve medicine adherence for patients , as well as the use of technology for adverse event monitoring, decentralised clinical trials and—where appropriate—the reuse and recycling of unused medications.

Waste represents another challenge and opportunity for care in the community, with the NHS in England alone producing approximately 156,000 tonnes of clinical waste each year^12^ Shifting more care into the community and greater use of HaH will require a recalibration and consideration of best practice for how, where and when this waste will be collected. This change may also provide an opportunity to embrace technology and innovation to explore ways to reduce inefficiencies and embrace principles of circularity.

### Hardware, data and infrastructure

Whilst digital innovation and diagnostics provide many opportunities to improve healthcare, technology-enabled care itself has an inherent and often significant environmental footprint^23,24^. A greater reliance on digital and data to enable a shift from hospital care to HaH will therefore require a reconsideration of the potential negative impacts of digital infrastructure, data and hardware including mitigations. This is particularly important regarding the enabling technologies for remote patient monitoring, point of care diagnostics and the interoperability necessary to articulate with existing electronic health record systems and infrastructure. Closer consideration should also be given to how devices used for remote patient monitoring are procured (including embodied carbon), deployed, and reused. In particular, research suggests that there are high rates of ‘hibernating’ devices of consumer technology where devices no longer being used are hindering the recycling and circular economy of wearables and mobile devices^25–27^. Other factors to consider include the materials or carbon footprint of the hardware used, which can encompass the materials used, their durability, energy charging capacity, and efficiency^26^. Moreover, there is an opportunity to consider the principles of reduce, reuse and recycle within diagnostics, hardware and technology—for example, by considering the minimum required resolution for scans, images and videos to avoid unnecessary data processing and storage.

### Food

Although not traditionally considered in the remit of HaH services, the social determinants of health, including diet, have a significant impact on our health, economy, and the environment. Here, we propose food be included in discussions as a key point of interest moving forward. In 2021, the NHS is estimated to have spent £6.5 billion on obesity-related ill health^28^. At the same time, the NHS in England provides 140 million in-patient meals per year costing £633 million^29^, and is responsible for an estimated 1543 kilotons of CO_2_ equivalent annually, accounting for around 6% of the NHS’s overall emissions^29,30^. Therefore opportunities exist to potentially mitigate this environmental impact while simultaneously promoting public health. One solution already proposed is to encourage the adoption of healthier diets such as seasonal fruits and vegetables, legumes, pulses, and alternative protein sources with lower environmental footprints^31^. However, this carries a number of considerations and complexities to make it available equitably including cost, practicality of implementation, and acceptability. Overall, shifting more care into the community and hospital at home will require close consideration, engagement and evaluation of the role of food interacts with existing provision and funding models. This may include where innovation and technology may be able to support (e.g., Personalised nutrition advice, meal planning, remote monitoring, and delivery services).

## Considerations for the future and potential next steps

### Towards a systems-based assessment of co-benefits

**Fig. 3:**
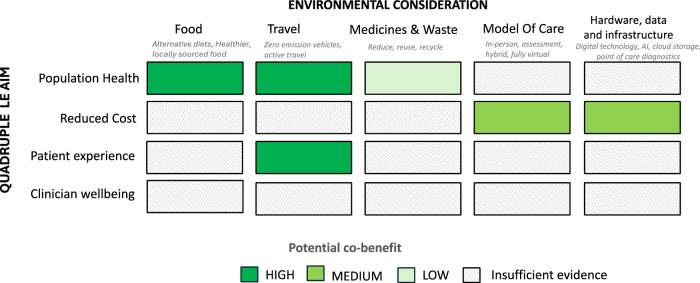
Systems based assessment of co-benefits. A proposed systems based framework for assessment for future research: Comparing the Quadruple Aim of Healthcare (x axis) with specific environmental considerations (y axis). With potential co-benefits highlighted according to estimated strength of evidence.

Now more than ever, balancing the environmental, economic and population health will require a firm evidence base to begin careful prioritisation of choices and investments. This includes evidence on adaptation, mitigation and the holistic assessment of systems’ capability to cater for multimorbidity and to adapt to the geographical and place-based challenges and opportunities. The generation of this evidence will require harmonising assessment methodologies that are often currently siloed (e.g economic versus environmental research) and instead focussing on ‘multimodal’ assessment. In Fig. 3 we propose a potential framework to begin the discussion on assessing the trade-offs, and interactions between environment and quintuple aims of healthcare.

### Focus on prevention and ‘Health@Home’

It could be argued the most environmentally friendly hospital visit is the one you can prevent or avoid. As such healthcare system transformations favouring disease prevention and chronic disease management have the potential for dual reductions: lower environmental impact and improving health outcomes. There is therefore opportunity to explore how HaH can be used in a more preventative way, expanding the scope of traditional HaH functions outlined in Fig. 1.

### Adapt to changing workforce and skills

Achieving a net-zero health system will depend on closing gaps in knowledge and skills for health professionals. To provide healthcare in a more sustainable way, clinical staff need a tailored set of skills that align with their existing professional development plans. A vital step is to raise the awareness of clinicians and patients about the environmental impact of current care pathways and services and how they can make a positive difference with their actions. This can also involve working more closely with patients to design greener approaches to care. Lastly, ongoing monitoring of the impact of these changes and sharing best practices across teams and organisations is essential for enhancing positive environmental outcomes.

## Conclusion

Emerging healthcare delivery models like HaH hold promise as a solution to the growing complexities of population health. Despite climate change now a key consideration and factor in health, the environmental considerations of HaH, including its integration with existing systems has been relatively unexplored. Building a robust evidence base is essential for making informed decisions and prioritise investments that optimise environmental sustainability, economic viability, and population health outcomes, while mitigating existing health disparities. Importantly, It may be the most environmentally friendly hospital visit is the one you can prevent or avoid therefore HaH’s potential for preventive care needs further exploration, which might involve harnessing innovation, technology, and workforce development. As these models of care continue to develop, it is crucial to conduct more holistic evaluations that assess their economic, social, health, and environmental goals.

## References

[1] Alami, H., Lehoux, P., Miller, F. A., Shaw, S. E. & Fortin, J. P. An urgent call for the environmental sustainability of health systems: A ‘sextuple aim’ to care for patients, costs, providers, population equity and the planet. *Int. J. Health Plan. Manag.* **38**, 289–295 (2023). 10.1002/hpm.3616 36734815

[2] Pandit, J. A. et al. The hospital at home in the USA: current status and future prospects. *npj Digit. Med.* **7**, 48 (2024). 38413704 10.1038/s41746-024-01040-9 PMC10899639

[3] Knight, T. et al. The provision of hospital at home care: Results of anational survey of UK hospitals. *Int. J. Clin. Pract.* **75**, e14814 (2021). 34510673 10.1111/ijcp.14814

[4] Norman, G., Bennett, P. & Vardy, E. R. L. C. Virtual wards: a rapid evidence synthesis and implications for the care of older people. *Age and Ageing* **52**, 1–12 (2023). 10.1093/ageing/afac319 PMC9835137 36633298

[5] Arsenault-Lapierre, G. et al. Hospital-At-home interventions vs in-hospital stay for patients with chronic disease who present to the emergency department. *JAMA Netw. Open* **4**, E2111568 (2021). 34100939 10.1001/jamanetworkopen.2021.11568 PMC8188269

[6] The Health Foundation. How do the public and NHS staff feel about virtual wards? https://www.health.org.uk/news-and-comment/charts-and-infographics/how-do-the-public-and-nhs-staff-feel-about-virtual-wards (2023).

[7] NICE. *Virtual Wards as Alternatives to Hospital Care Economic Evidence Review*. https://view.officeapps.live.com/op/view.aspx?src=https%3A%2F%2Fwww.nice.org.uk%2FMedia%2FDefault%2FAbout%2Fwhat-we-do%2FHTA%2520Lab%2Fvirtual-wards-economic-evidence-review.docx&wdOrigin=BROWSELINK (2023).

[8] Health Care Without Harm. Health care climate footprint report | Health Care Without Harm. https://noharm-global.org/documents/health-care-climate-footprint-report (2019).

[9] NHS. Delivering a ‘Net Zero’ National Health Service. (2020).

[10] NHS England. Artificial intelligence to help boost NHS winter response and prevent avoidable admissions. https://www.england.nhs.uk/2023/11/artificial-intelligence-to-help-boost-nhs-winter-response-and-prevent-avoidable-admissions/.

[11] Greener NHS. Greener NHS» Areas of focus. https://www.england.nhs.uk/greenernhs/a-net-zero-nhs/areas-of-focus/.

[12] NHS England. NHS clinical waste strategy. https://www.england.nhs.uk/publication/nhs-clinical-waste-strategy/.

[13] Wolf, R. M. et al. Potential reduction in healthcare carbon footprint by autonomous artificial intelligence. *npj Digital Med.* **5**, 1–4 (2022). 10.1038/s41746-022-00605-w PMC9098499 35551275

[14] Waterall, J., Rhodes, D. & Exley, K. Why air pollution is an important issue for all nurses. *Br. J. Nurs.* **30**, 982–983 (2021). 34514830 10.12968/bjon.2021.30.16.982

[15] GOV UK. Air pollution: applying All Our Health - GOV.UK. *GOV.UK* https://www.gov.uk/government/publications/air-pollution-applying-all-our-health/air-pollution-applying-all-our-health.

[16] Evaluation of using AutoPlanner in Community Nursing teams - SCFT Digital. https://digitaltransformation.scft.nhs.uk/2022/04/14/evaluation-of-using-autoplanner-in-community-nursing-teams/.

[17] NHS England. Patient self-management of inflammatory bowel disease with a smartphone app - Gastroenterology digital playbook - NHS Transformation Directorate. https://transform.england.nhs.uk/key-tools-and-info/digital-playbooks/gastroenterology-digital-playbook/patient-self-management-of-inflammatory-bowel-disease-with-a-smartphone-app/.

[18] Stuber, J. M. et al. Real-world nudging, pricing, and mobile physical activity coaching was insufficient to improve lifestyle behaviours and cardiometabolic health: the Supreme Nudge parallel cluster-randomised controlled supermarket trial. *BMC Med.* **22**, 1–15 (2024). 38303069 10.1186/s12916-024-03268-4 PMC10835818

[19] 22/102 HSDR Supporting the delivery of net zero health and social care system - supporting information | NIHR. https://www.nihr.ac.uk/documents/21577-hsdr-supporting-the-delivery-of-net-zero-health-and-social-care-system-supporting-information/29180.

[20] Aljofan, M. et al. The rate of medication nonadherence and influencing factors: A systematic Review. *Electron. J. Gen. Med.* **2023**, 2516–3507 (2023).

[21] Stewart, S.-J. F., Moon, Z. & Horne, R. Medication nonadherence: health impact, prevalence, correlates and interventions. (2022) 10.1080/08870446.2022.2144923. 10.1080/08870446.2022.2144923 36448201

[22] Brown, M. T. & Bussell, J. K. Medication Adherence: WHO Cares? *Mayo Clin. Proc.* **86**, 304 (2011). 21389250 10.4065/mcp.2010.0575 PMC3068890

[23] Samuel, G., Anderson, G. M., Lucivero, F. & Lucassen, A. Why digital innovation may not reduce healthcare’s environmental footprint. *BMJ***385**, e078303 (2024).38830688 10.1136/bmj-2023-078303

[24] Usher, K., Williams, J. & Jackson, D. The potential of virtual healthcare technologies to reduce healthcare services’ carbon footprint. *Front Public Health* **12**, 1394095 (2024). 38818441 10.3389/fpubh.2024.1394095 PMC11137209

[25] Powell, D. & Godfrey, A. *Considerations for Integrating Wearables into the Everyday Healthcare Practice*. *npj Digital Medicine* vol. 6 1–3 (Nature Publishing Group, 2023). 10.1038/s41746-023-00820-z PMC10122642 37087520

[26] Wilson, G. T. et al. The hibernating mobile phone: dead storage as a barrier to efficient electronic waste recovery. *Waste Manag.* **60**, 521–533 (2017). 28063833 10.1016/j.wasman.2016.12.023

[27] Inghels, D. & Bahlmann, M. D. Hibernation of mobile phones in the Netherlands: the role of brands, perceived value, and incentive structures. *Resour. Conserv. Recycl.* **164**, 105178 (2021). 10.1016/j.resconrec.2020.105178

[28] Department of Health and Social Care. Government plans to tackle obesity in England. https://healthmedia.blog.gov.uk/2023/06/07/government-plans-to-tackle-obesity-in-england/ (2023).

[29] ERIC. Estates Returns Information Collection - Summary page and dataset for ERIC 2020:21 - NHS Digital. (2021).

[30] NHS England» Food and nutrition. https://www.england.nhs.uk/ahp/greener-ahp-hub/specific-areas-for-consideration/food-and-nutrition/.

[31] Laine, J. E. et al. Co-benefits from sustainable dietary shifts for population and environmental health: an assessment from a large European cohort study. *Lancet Planet Health* **5**, e786–e796 (2021). 34688354 10.1016/S2542-5196(21)00250-3 PMC8581185

